# Randomized Comparison of Selective Internal Radiotherapy (SIRT) Versus Drug-Eluting Bead Transarterial Chemoembolization (DEB-TACE) for the Treatment of Hepatocellular Carcinoma

**DOI:** 10.1007/s00270-014-1012-0

**Published:** 2014-11-07

**Authors:** Michael B. Pitton, Roman Kloeckner, Christian Ruckes, Gesine M. Wirth, Waltraud Eichhorn, Marcus A. Wörns, Arndt Weinmann, Mathias Schreckenberger, Peter R. Galle, Gerd Otto, Christoph Dueber

**Affiliations:** 1 Department of Diagnostic and Interventional Radiology, Johannes Gutenberg University Medical Center, Langenbeckstr. 1, 55131 Mainz, Germany; 2 IZKS, Johannes Gutenberg University Medical Center, Langenbeckstr. 2, 55131 Mainz, Germany; 3 Department of Nuclear Medicine, Johannes Gutenberg University Medical Center, Langenbeckstr. 1, 55131 Mainz, Germany; 4 Department of Internal Medicine, Johannes Gutenberg University Medical Center, Langenbeckstr. 1, 55131 Mainz, Germany; 5 Department of Transplantation Surgery, Johannes Gutenberg University Medical Center, Langenbeckstr. 1, 55131 Mainz, Germany

**Keywords:** Selective internal radiotherapy (SIRT), Drug-eluting bead-transarterial chemoembolization (DEB-TACE), Radiation induced liver disease (RILD), Liver cirrhosis

## Abstract

**Purpose:**

To prospectively compare SIRT and DEB-TACE for treating hepatocellular carcinoma (HCC).

**Methods:**

From 04/2010–07/2012, 24 patients with histologically proven unresectable N0, M0 HCCs were randomized 1:1 to receive SIRT or DEB-TACE. SIRT could be repeated once in case of recurrence; while, TACE was repeated every 6 weeks until no viable tumor tissue was detected by MRI or contraindications prohibited further treatment. Patients were followed-up by MRI every 3 months; the final evaluation was 05/2013.

**Results:**

Both groups were comparable in demographics (SIRT: 8males/4females, mean age 72 ± 7 years; TACE: 10males/2females, mean age 71 ± 9 years), initial tumor load (1 patient ≥25 % in each group), and BCLC (Barcelona Clinic Liver Cancer) stage (SIRT: 12×B; TACE 1×A, 11×B). Median progression-free survival (PFS) was 180 days for SIRT versus 216 days for TACE patients (*p* = 0.6193) with a median TTP of 371 days versus 336 days, respectively (*p* = 0.5764). Median OS was 592 days for SIRT versus 788 days for TACE patients (*p* = 0.9271). Seven patients died in each group. Causes of death were liver failure (*n* = 4 SIRT group), tumor progression (*n* = 4 TACE group), cardiovascular events, and inconclusive (*n* = 1 in each group).

**Conclusions:**

No significant differences were found in median PFS, OS, and TTP. The lower rate of tumor progression in the SIRT group was nullified by a greater incidence of liver failure. This pilot study is the first prospective randomized trial comparing SIRT and TACE for treating HCC, and results can be used for sample size calculations of future studies.

## Introduction

Hepatocellular carcinoma (HCC) is one of the most common cancers with an annual incidence of around 750.000 per year worldwide [1, 2]. Its incidence is still rising, mainly due to the increasing numbers of Hepatitis B (HBV) and C virus (HCV) infections [3]. Unfortunately, the majority of patients are diagnosed in intermediate or advanced clinical stages excluding them from potentially curative treatments like resection, transplantation, or local ablation. According to the barcelona clinic liver cancer classification (BCLC), patients with intermediate stage HCC (BCLC stage B) should undergo transarterial chemoembolization (TACE) [1, 4, 5]. In intermediate stage HCC, TACE has a proven survival benefit compared to the best supportive care with 1- and 2-year survivals of 82 and 63 %, respectively [6]. Precision-V study has defined a standardized embolization technique with drug eluting beads compared to the variety of different conventional TACE protocols reported in the literature [7, 8].

During the last few years, selective internal radiotherapy (SIRT), also referred to as radioembolization, was introduced for HCC as a second-line therapy in case of TACE failure. One of its main advantages is the reduced number of treatments needed; therefore, SIRT has potential as a first-line therapy for patients with intermediate stage HCC (BCLC stage B) despite its slightly higher costs [9]. Our impression is that the reduced number of treatment sessions and the small size of the embolization particles preserve patency of the tumor feeding arteries. Since this maintains direct access to the tumor vessels, another local treatment, e.g., TACE, could still be performed as a second-line treatment in case of SIRT failure. Randomized data for HCC treatment using SIRT are not currently available. The only reports on treatment results are retrospective [10–12], non-randomized [13], or deal with feasibility of treatment in advanced HCC, dose-finding, and SIRT-associated complications [14]. The aim of this randomized pilot study was to investigate whether SIRT might compare with, or even have some advantages over, TACE for treating HCC.

## Patients and Methods

### Study Design

This study was a prospective, single-center, randomized trial with two parallel treatment groups receiving either DEB-TACE or SIRT. The trial was conducted on the basis and principles of ICH-GCP and according to the Declaration of Helsinki in its revised version. The study protocol (including patient information with consent) and any substantial amendments were approved by the responsible Ethics Committee. The study is registered at www.clinicaltrials.gov as NCT01798160. A literature review in 2010 provided papers on SIRT-associated complications [15], feasibility of SIRT in advanced patients [16], and dose finding. The only studies describing the treatment results of SIRT are retrospective [17] or non-randomized [13] and investigated an inhomogeneous patient collective not comparable to stage B patients according to BCLC [4]. Therefore, we finally decided that a reliable power calculation is not feasible and label this study a pilot study.

### Patients

The final study population consisted of 24 patients, 12 in each group of patients (mean age in SIRT group 71.8 ± 7.2 years, range, 58–82 years; mean age in TACE group 70.5 years, range 59–87 years). The SIRT group consisted of eight men and four women, the TACE group comprised ten men and two women. All patients suffered from histologically proven M0 N0 HCC. The indication for local tumor treatment was assessed by an interdisciplinary HCC-tumor board. All patients meeting the inclusion criteria and eligible for both SIRT and TACE were included (Table 1). Informed consent was obtained from all individual participants included in the study. After stratification according to tumor load (<25 %/≥25 %), patients were randomized. Treatment allocation was predetermined by an independent statistician and used a randomized block design. The final randomization was carried out after having obtained written informed consent.

**Table 1:** Inclusion/exclusion criteria

Inclusion criteria
≥18 years
HCC, proven by histology or according to EASL criteria
Intermediate stage HCC (stage B according to BCLC)
At least one measurable lesion in magnetic resonance imaging (MRI)
Tumor load ≤50 %
Preserved liver function (Child Pugh A – B7)
Exclusion criteria
Patients feasible for curative treatment (e.g., resection or local ablation)
Previous TACE or SIRT
Chemotherapy during the last 4 weeks
Child Pugh stage C
BCLC stage C
ECOG Performance Status >0
Tumor involvement >50 % of the liver
Extrahepatic tumor
Serum bilirubin >2.0 mg/dl; serum albumin 2.8 g/dl, serum creatinine >2 mg/dl; leukocytes <3,000/ml; thrombocytes <50,000/ml
Clinically apparent ascites (ascites only in CT/MRI is no exclusion criteria)
Esophageal bleeding during the last 3 months
Hepatic encephalopathy
Transjugular intrahepatic portosystemic shunt (TIPS)
Infiltration or occlusion of the portal vein
Hepatopulmonary shunt ≥20 % in the macroaggregated albumin (MAA) scan
Contraindications against angiography
Gravidity

### Liver MRI

Liver imaging was performed with contrast enhanced MRI using 3-Tesla scanners (Skyra^®^ or Magnetom Trio^®^, Siemens). Study protocols included: T1w FLASH 2d (in- and opposed phase), T2w HASTE, DWI, contrast enhanced dynamic 3d VIBE sequences [start delay after contrast bolus: 0 s (native), 20 s (arterial), 45 s (portal-venous), and 90 s (equilibrium-Phase)], and a late 2d FLASH phase. Gadolinium-DTPA (Magnevist^®^, Bayer Schering Pharma AG), 0.1 mmol/kg body weight, 2 ml/s, was administered by bolus injection (Spectris^®^, Medrad) for contrast. Image analysis was carried out independently by two investigators experienced in cross sectional liver imaging. This analysis was followed by a consensus reading to obtain the final diagnosis.

### CT Scan

To rule out extrahepatic tumor spread, all patients underwent CT of the thorax and abdomen (256-row iCT^®^, Philips Medical Systems) with standard acquisition parameters: abdomen: 120 kV, mAs according to automatic dose modulation, slice thickness 1 and 3 mm, contrast bolus 120 ml Iomeprol (Imeron 400^®^ Altana Pharma) using a bolus injector (Injektron CT2^®^, Medtron) with a flow rate of 4 ml/s, and a start delay by bolus trigger 150 HE—native, 10 s (arterial) and 45 s (portal-venous); thorax: 120 kV, mAs according to automatic dose modulation, slice thicknesses 1 and 3 mm.

### SIRT Procedure

The SIRT procedure has been extensively described [18]. Patients underwent preparative intervention with angiography of the hepatic artery and protective coiling of side branches (e.g., gastroduodenal artery, right gastric artery). Afterwards, 150 MBq ^99m^Tc-MAA was injected into the liver arteries using the same catheter position chosen for the scheduled SIRT session. Calculation of the hepato-pulmonary shunt fraction and tracer distribution was evaluated with subsequent planar images and SPECT imaging (MAA-scan) [14, 16, 19]. Patients were then discharged and re-admitted for SIRT.

SIRT was performed using resin-based ^90^Y loaded microparticles (SirSpheres^®^, Sirtex Medical). The activity and dose for ^90^Y-SirSpheres^®^ were calculated according to the body surface model as suggested by the REBOC expert panel [20, 21]. SIRT was performed in a lobar approach. In case of bilobar tumor spread, treatment was split in two sessions. In these cases, the first treatment was dedicated to the liver lobe with the greater tumor volume. Treatment of the contra-lateral lobe was scheduled after 4 weeks to preserve liver function. After each treatment, patients were monitored for 2 days. Follow-up visits were performed every 3 months until clinical endpoints were reached. In cases with local tumor progression and an absence of contraindications, SIRT could be repeated once according to the study protocol. In cases with contraindications, crossover to TACE was permitted. Patients with crossover from SIRT to TACE were not censored.

### TACE Procedure

TACE was performed using drug-eluting beads (DC Beads^®^, 100-300 µm, Terumo) loaded with a maximum dose of 150 mg Doxorubicin per session [22]. The beads were administrated super selectively at the level of segmental and subsegmental arteries until stasis was reached (embolization endpoint). In patients with multilocular tumor spread or bilobar disease preventing a selective approach, a less selective embolization technique was used and each session was limited to one liver lobe according to the discretion of the investigator. In those cases, the contra-lateral lobe was treated after 4 weeks. TACE was repeated every 6 weeks until no more viable tumor was detected by MRI [7, 23]. Then, follow-up visits were performed every 3 months until clinical endpoints were reached. In cases with local tumor progression and an absence of contraindications, TACE could be repeated. If contraindications appeared, crossover to SIRT was possible according to the protocol. Patients with crossover from TACE to SIRT were not censored. Follow-up was carried out every 3 months and included physical examination, blood tests, documentation of adverse-/serious adverse-events (AE/SAE), and MRI of the liver.

### Outcome Measures

Survival is not only limited by tumor progression, but also by deterioration of liver function as a result of the underlying liver cirrhosis. Therefore, the primary endpoint was progression-free-survival (PFS) as it includes local treatment effects as well as death by deteriorating liver function [5, 24]. Local tumor response was measured according to the modified criteria for response evaluation in solid tumors (mRECIST) [25]. Overall survival (OS) is also influenced by secondary treatment strategies following SIRT/TACE failure; therefore, it was applicable as a secondary endpoint. Time to progression (TTP) and time-to-locally-non-treatable-progression (nTTP) were also calculated. The TTP was defined as the time at which progression was evident according to the definitions of mRECIST in follow-up MRI or CT (extrahepatic disease). The nTTP was defined as the time at which local tumor progression of the liver could no longer be treated with the assigned treatment regime (SIRT or TACE) because of contraindications (e.g., laboratory findings), technical problems (e.g., occluded access artery), and/or occurrence of an extrahepatic tumor, preventing a continued local treatment approach.

The causes of deaths were analyzed with respect to the clinical aspect of the patients, laboratory tests, and imaging data. Death due to tumor progression was determined when tumor load was considerably increased, with replacement of normal liver tissue and/or invasion of large vessels possibly associated with liver failure and/or significant extrahepatic tumor spread. Vice versa, death due to liver failure was determined when liver function decreased (e.g., extensive ascites and need for paracentesis, increasing bilirubin levels, and impaired coagulation status) in the absence of an increasing tumor load.

### Statistical Analysis

Statistical analysis system (SAS^®^), Version 9.2 (SAS Institute Inc.) was used for analysis, which was purely exploratory. Statistical analysis was done by an independent statistician to avoid review bias. Primary and secondary outcome measures (PFS, OS, TTP, and nTTP) were compared using the log-rank test. Time-to-event data were analyzed by the Kaplan–Meier method and descriptive statistics of all other parameters were provided. The null hypothesis was that there is no difference in PFS for intermediate stage HCC patients treated with DEB-TACE or SIRT.

## Results

### Patient Characteristics

**Fig. 1:**
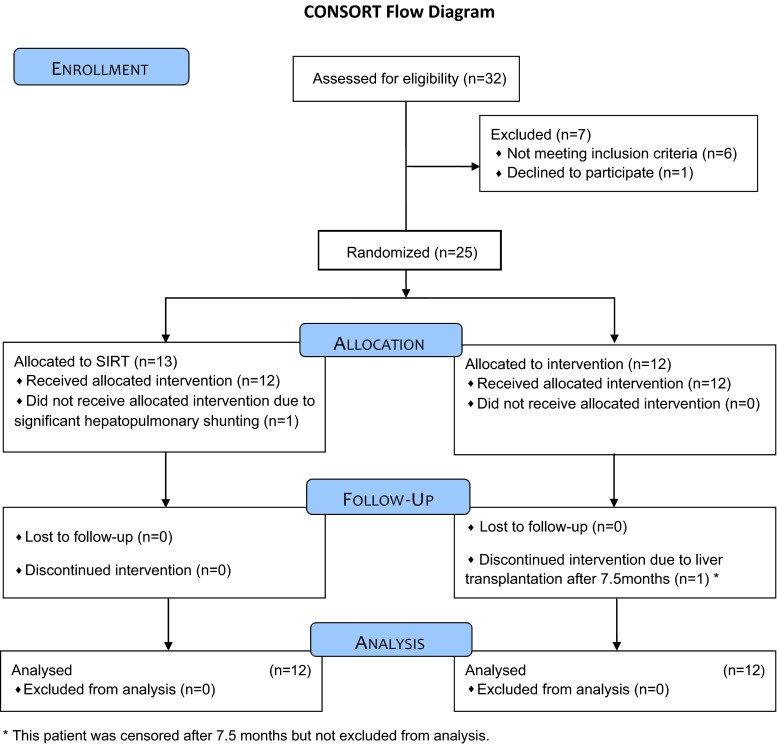
Flowchart according to the CONSORT guidelines

Between April 2010 and July 2012, thirty-two patients with intermediate stage HCC were screened for study inclusion. Seven of these patients were excluded due to poor liver function (*n* = 3), extrahepatic tumor spread in CT (*n* = 3), and withdrawal of consent immediately before randomization (*n* = 1). Subsequently, 25 patients were randomized, 13 to SIRT and 12 to TACE treatment. One SIRT patient showed significant hepatopulmonary shunting in the MAA scan and had to be excluded from treatment. Thus, 12 patients were treated in each group. There were no cases lost to follow up. One patient in the TACE group underwent liver transplantation 7.5 months after randomization and was, therefore, censored (Fig. 1). At study entry, there were no significant differences between groups in patients’ ages and genders, or liver function (Table 2). The size of target lesions according to mRECIST was not significantly different between both groups (SIRT: 61.3 ± 36.4 mm, range 10–134 mm; TACE: 60.8 ± 37.6 mm, range 30–163 mm), nor was the distribution of tumors in the liver (uni-/bilobar: SIRT 4/8; TACE 5/7), or the distribution of tumor volume (one patient with ≥25 % tumor load in each group; Table 2).

**Table 2:** Patient characteristics and treatment strategy

Patient demographics	SIRT	TACE
Patients treated (n)	12	12
Male/Female	8/4	10/2
Age (years)	71.8 ± 7.2 (58–82)	70.5 ± 9.0 (59–87)
Etiology of liver cirrhosis		
(alcohol/HCV/HBV/cryptogen)	5/5/0/2	5/4/1/3*
Prior curative treatment		
Resection/local ablation	3/4	5/1
Tumor burden		
SLD** (mm)	61.3 ± 36.4 mm (10–134)	60.8 ± 37.6 mm (30–163)
Tumor volume < 25 %/≥ 25 %	11/1	11/1
Tumor grading		
G1/G2/G3	6/6/0	6/5/1
AFP (ng/ml)	3308 ± 10204 (6.2–32346***) Median 14.0	164 ± 529 (2.7–1847***) Median 7.8
Liver function		
Child A/B/C	10/2/0	9/3/0
BCLC A/B	0/12	1/11
Laboratory		
Bilirubin (mg/dl)	1.17 ± 0.54 (0.38–2.10)	1.26 ± 0.55 (0.59–2.04)
Albumin (g/l)	34.08 ± 5.57 (28–43)	31.92 ± 4.25 (24–39)
INR	1.11 ± 0.12 (1–1.4)	1.13 ± 0.09 (1–1.3)
Thrombocytes (/nl)	159.83 ± 53.59 (111–265)	156.25 ± .85.03 (59–402)
Leucocytes (/nl)	5.23 ± 1.60 (2.27–8.28)	5.49 ± 1.52 (3.96–8.20)
Treatment strategy		
Randomization to treatment (days)	28.8 ± 13.8 (13–56)	15.7 ± 5.9 (4–24)
Treatment session per patient (n)	1.5 ± 0.5 (1–2)	3.8 ± 2.6 (1–10
Interval between treatment sessions (days)	33.5 ± 6.8 (27–42)	48.2 ± 14.0 (19–89)
Uni-/bilobar approach	4/8	5/7
Dose		
Total liver dose	1847 ± 504 MBq (1160–2940)	259.4 ± 158.4 mg (87.5–648.5)
Right liver lobe dose	1216 ± 288 MBq (830–1630)	205.4 ± 76.5 mg (144.5–359.5)
Left liver lobe dose	946 ± 250 MBq (590–1460)	126.4 ± 68.4 mg (60–289)
Follow-up (days)	435 ± 320 (77–1024)	404 ± 304 (52–950)

Data given as mean ± SD (range)

* One patient with HBV/HCV co-infection

** SLD sum of longest diameters of target lesions according to mRECIST

*** Extensive AFP level in one patient in each group

### SIRT/TACE procedures and follow-up

SIRT was performed selectively in a lobar approach. SIRT patients received only one (*n* = 4) or two treatment sessions (*n* = 8), with 33.5 ± 6.8 days between sessions, according to the protocol. The activity of ^90^Y-SirSpheres^®^ was 1847 ± 504 MBq, with a wide range depending on tumor volume and liver function. In TACE patients, the mean number of treatment sessions was 3.8 ± 2.6 with a range of 1–10 depending on local tumor response and patency of the access vessels. The interval between subsequent treatment sessions was 48.2 ± 14.0 days. Embolization was unilobar in five and bilobar in seven patients, depending on the tumor distribution and local tumor response evaluated by MRI. The cumulative Doxorubicin dose was 259.4 ± 158.4 mg with a 150 mg maximum of Doxorubicin-loaded DC Beads^®^ per treatment session. Follow-up was 435 ± 320 days for SIRT and 404 ± 304 days for TACE and included all events until the final evaluation date (Table 2).

### Outcome

**Fig. 2:**
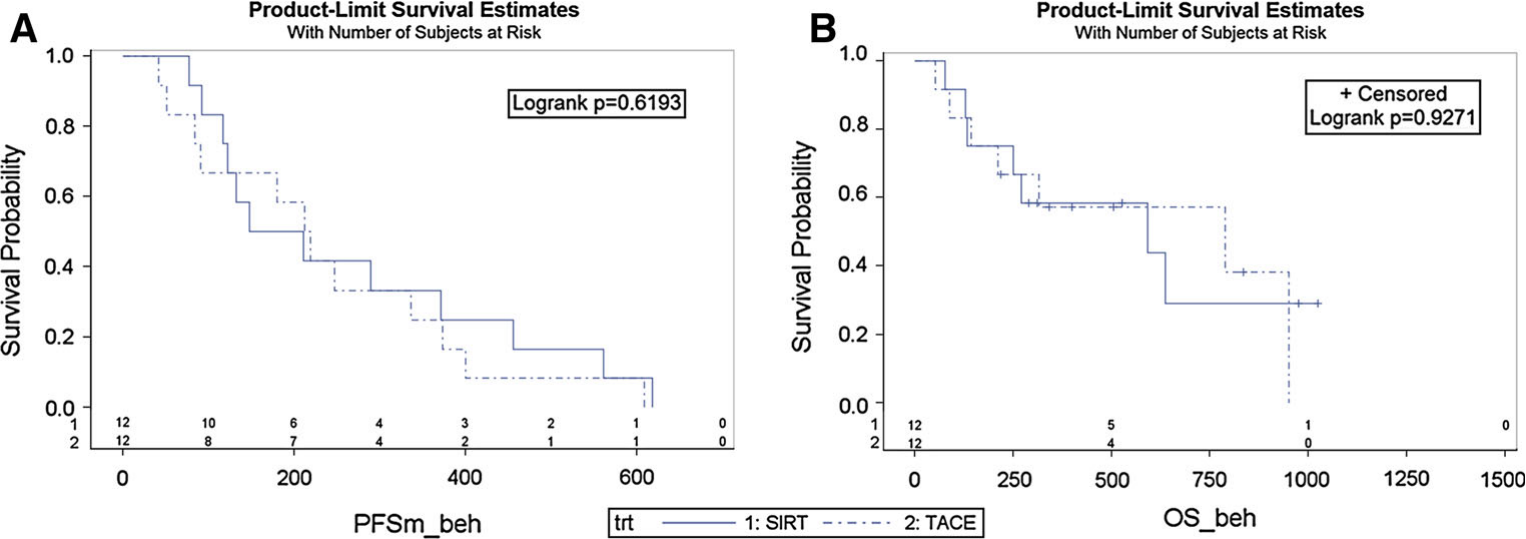
**A** Progression-free-Survival (PFS) in days (Progression: PD according to mRECIST): Treatment (trt) *1* SIRT, Treatment (trt) *2* TACE **B** Overall Survival. Treatment (trt) *1* SIRT, treatment (trt) *2* TACE

There were no statistically significant differences between both groups in PFS (180 for SIRT versus 216 days for TACE, Fig. 2A) and OS (592 days for SIRT vs. 788 days for TACE, Fig. 2B; Table 3). There was no 30-day mortality in either group. During follow-up, mortality after SIRT was caused predominantly by liver failure (*n* = 4) with only one death due to tumor progression. In the TACE group, four patients died from tumor progression and only one due to liver failure. One patient in each group died from a cardiovascular complication. One patient presented with progressive heart failure and edema 3 months after the first TACE. She did not receive repeated TACE because of an arteriovenous shunt and substantial tumor progression after the one session. Another patient was re-admitted 7 days after SIRT. She suffered from a pseudoaneurysm of the femoral artery (access site complication) and a large retroperitoneal hematoma. The access site was surgically revised and the hematoma was released. Subsequently, she developed renal insufficiency and heart failure with the need for hemodialysis. After several days of dialysis, she refused continued treatment and died 42 days after SIRT. For one patient in each group, there was non-conclusive data on the final cause of death.

**Table 3:** Outcome measures

Outcome measures	SIRT	TACE	*p*
PFS	180 (120/414); 266 ± 55	216 (88/355); 237 ± 49	*p* = 0.6193
OS	592 (192/–); 437 ± 72	788 (178/950); 583 ± 119	*p* = 0.9271
TTP	371 (132/561); 353 ± 69	336 (91/609); 315 ± 69	*p* = 0.5764
nTTP	488 (148/925); 490 ± 114	647 (182/–); 416 ± 83	*p* = 0.9322

The median number of days (Q1/Q3) and mean ± standard errors are shown

*PFS* Progression-free-Survival, *OS* overall-survival, *TTP* time-to-Progression, *nTTP* time-to-non-treatable-progression

There was no significant difference between groups with respect to TTP (371 days after SIRT versus 336 days after TACE) and nTTP (488 after SIRT versus 647 days after TACE) (Table 3). In the SIRT group, no patient had a repeated SIRT treatment because of contraindications. Three of four patients with local tumor progression developed conditions that predicted liver insufficiency (new ascites and elevated bilirubin levels >2.0 mg/dl), which prevented repeated SIRT. These patients received DC-Bead TACE, as the bilirubin levels were <3 mg/dl and no contraindications for TACE were evident. Another patient with local tumor progression developed an arterioportal shunt, which was a contraindication for SIRT and TACE.

In the TACE group, 8 out of 12 patients presented with diverse changes of the tumor feeding arteries. As the number of TACE procedures increased (median 3.5, range 1–10), these patients presented with increasing irregularities and narrowing of vessel walls, or even occlusions. Bypassing and insufficient reticular networks of collateral vessels prevented selective access to the tumor nodules and application of a sufficient embolization dose. In five of these eight patients, repeated treatment had to be aborted. In addition, there was one patient with an iatrogenic dissection of the celiac trunk that prohibited further catheterization and two patients with arteriovenous shunts with a myriad of crosslinks preventing all embolization techniques.

After cessation of loco-regional treatment, one patient received curative liver transplantation (TACE group) and two received local tumor ablation, one in each group (Table 4). The patient who underwent liver transplantation was censored for further statistical calculations. The other patients with tumor progression received Sorafenib, local radiation of critical bone metastases, and underwent clinically indicated surgeries (Table 4).

**Table 4:** Causes of death and clinical events

Causes of death	SIRT	TACE
Tumor progression	1	4
Liver failure	4	1
Cardiovascular event	1	1
Non-conclusive	1	1
Suspension from randomized treatment strategy		
Access vessel vanishing		3 (5*)
Dissection of access vessel		1
Arteriovenous fistula due to local tumor progression	1	2
Thrombosis of the main portal vein		1
Local tumor progression		2
Local tumor progression and ascites	3	
Complications of access vessels		
Damage to arterial feeding vessel		8
Vanishing after 1–5 treatment sessions (median 3.5)		7
Dissection of access vessel		1
Tumor treatment beyond randomized treatment		
Liver transplantation		1
Microwave-ablation	1	1
DC-Bead-TACE	3	
Radiation of spine metastases/radiation and spondylodesis	1/1	1/0
Systemic treatment with Sorafenib	3	5
Significant clinical findings		
Arterioportal and arteriovenous fistulas by tumor progression	1	2
Bone metastases	2	2
Lung metastases		1
Portal vein thrombosis	2	2
Liver vein thrombosis		1
Liver insufficiency	5	2
Encephalopathy	1	
Variceal bleeding/bleeding and ligation of esophageal varices	1/0	2/1
Angina pectoris and aortocoronary-bypass-graft		1
False aneurysm of the femoral artery (access site), surgical revision with subsequent renal failure and hemodialysis	1	

* One case without vascularized tumor under surveillance and without need for further embolization treatment, one patient with a favorable local response who died after a cardiovascular event following coronary bypass grafting

## Discussion

This randomized clinical pilot study compared the oncologic efficiency of SIRT and TACE in patients with unresectable HCC. The potential risk of vessel damage as a result of repetitive embolization in TACE seems to be considerably lower in SIRT due to its smaller particle sizes and the reduced number of treatment sessions. This, together with the much easier application in the right and left hepatic artery makes it very attractive for the patient compared to super-selective TACE procedures with longer intervention times and repeated hospital admissions. The study protocol was designed to provide data on local tumor response, treatment associated peri-interventional complications, and potential side effects concerning liver function and overall survival. The results of this study might provide potential study endpoints, sample sizes, and safety aspects for a confirmatory multicenter study. The inclusion and exclusion criteria reflect the spectrum of HCC patients at our University Hospital with some variation in tumor load (similarly distributed in both groups) and without impaired liver function. The TACE group served as the control according to the allocation rules of the BCLC scheme [4]. The relatively small final sample size of 24 (2 × 12) patients reflects a study limitation.

Current data suggested SIRT was a favorable second-line treatment for diverse tumors, including HCC, after failure of standard treatment regimens [6, 11]. The results of this pilot study demonstrate no differences in the PFS and OS after SIRT and TACE and no significant statistical differences in TTP and nTTP. The median survival after TACE found in this study is comparable to reported survival data after DC-Bead TACE and represents typical clinical results [26]. However, the reduced number of SIRT treatment sessions and hospital days might be a significant difference that reflects an advantage in terms of quality of life [27]. Another potential advantage of SIRT over TACE is the significantly reduced harm caused to feeding arteries. The preserved patency of the tumor feeders allows for repeated transarterial tumor treatment with any kind of method in case of tumor progression. However, in the current study, patients with tumor progression presented with reduced liver function that prohibited repeated SIRT. A small number of cases crossed-over to TACE, but without benefit in overall survival. In addition, a considerable number of patients presented with occluded feeder arteries after repeated TACE that prevented direct transarterial access to the tumor and repeated embolizations of any kind.

We were able to differentiate between deaths due to progressive liver failure versus tumor progression. In all patients, the cause of death was identified while considering the clinical course. Death was due to liver failure when liver function decreased without a respective increase in tumor load and vice versa. The cause of deaths in this study might impact future interventional techniques. The less selective application of SirSpheres at the level of lobar arteries has the advantage of ease and speed of application; in addition, it is an effective treatment of non-visible micro-nodules that otherwise might become evident as recurrences during follow-up, which typically occurs for patients treated with super-selective TACE. However, there is an increased risk for secondary liver failure when one or both liver lobes are treated unselectively. Our data demonstrated secondary liver insufficiency as the primary cause of death after SIRT; while, there were considerably more deaths due to tumor progression in TACE patients. Radiation induced liver disease (RILD) has already been reported within 2 months following SIRT [14, 16, 21, 22]. In our patients, fatal liver insufficiencies occurred at 4, 8, 20, and 21 months after SIRT; however, it is not clear whether this was due to deterioration due to liver cirrhosis or to radiation induced toxicity beyond 2 months. Thus, further evaluations should examine whether a more selective SIRT application could reduce the incidence of liver failure. However, this would presumably result in a higher rate of local tumor progression in the non-treated liver segments, comparable to the super-selective TACE approach used. Nonetheless, this selective SIRT approach might require repetitive SIRT treatments.

In conclusion, this randomized pilot study suggests SIRT and TACE are equivalent in terms of progression-free survival, overall survival, and TTP in intermediate stage HCC patients. The lower rate of tumor progression in the SIRT group was nullified by a greater incidence of liver failure. The results of this analysis should aid future studies for clarifying treatment decisions, determining medical expenses and treatment costs, as well as supplementing the BCLC scheme.

## Abbreviations

SIRT: Selective internal radiotherapy
DEB-TACE: Drug-eluting bead-transarterial chemoembolization
HCC: Hepatocellular carcinoma
BCLC: Barcelona clinic liver cancer
PFS: Progression-free survival
TTP: Time to progression
OS: Overall survival
HBV: Hepatitis B virus
HCV: Hepatitis C virus
ICH: International conference on harmonisation of technical requirements for registration of pharmaceuticals for human use
GCP: Good clinical practice
FLASH: Fast Low Angle Shot
HASTE: Half fourier acquisition single shot turbo spin echo
DWI: Diffusion-weighted imaging
VIBE: Volumetric interpolated breath-hold examination
kV: Kilovoltage
mAs: Milliampere-seconds
99 mTc-MAA: 99m Technetium macroaggregated albumin
SPECT: Single photon emission computed tomography
AE/SAE: Adverse-/serious adverse-events
mRECIST: Modified criteria for response evaluation in solid tumors
nTTP: Time-to-locally-non-treatable-progression
RILD: Radiation induced liver disease
